# Near-Infrared Spectroscopy: A Free-Living Neuroscience Tool to Better Understand Diabetes and Obesity

**DOI:** 10.3390/metabo13070814

**Published:** 2023-07-03

**Authors:** Eleni Rebelos, Eleonora Malloggi, Martina Parenti, Angela Dardano, Andrea Tura, Giuseppe Daniele

**Affiliations:** 1 Department of Clinical and Experimental Medicine, University of Pisa, 56126 Pisa, Italy; eleni.rebelos@utu.fi (E.R.); eleonoramalloggi@gmail.com (E.M.); parentimartina3991@gmail.com (M.P.); angela.dardano@unipi.it (A.D.); 2 CISUP, Center for Instrument Sharing, University of Pisa, 56124 Pisa, Italy; 3 CNR Institute of Neuroscience, 35131 Padova, Italy; andrea.tura@cnr.it

**Keywords:** functional near-infrared spectroscopy, fNIRS, neuroimaging, obesity, type-2 diabetes

## Abstract

The human brain is the least accessible of all organs and attempts to study it in vivo rely predominantly on neuroimaging. Functional near-infrared spectroscopy (fNIRS) allows for the study of cortical neural activity in a non-invasive manner that may resemble free-living conditions. Moreover, compared to other neuroimaging tools, fNIRS is less expensive, it does not require the use of ionizing radiation, and can be applied to all study populations (patients suffering from claustrophobia, or neonates). In this narrative review, we provide an overview of the available research performed using fNIRS in patients with diabetes and obesity. The few studies conducted to date have presented controversial results regarding patients with diabetes, some reporting a greater hemodynamic response and others reporting a reduced hemodynamic response compared to the controls, with an unclear distinction between types 1 and 2. Subjects with obesity or a binge eating disorder have reduced prefrontal activation in response to inhibitory food or non-food stimuli; however, following an intervention, such as cognitive treatment, prefrontal activation is restored. Moreover, we discuss the potential of future applications of fNIRS for a better understanding of cortical neural activity in the context of metabolic disorders.

## 1. Introduction

The adoption of a westernized lifestyle, characterized by being sedentary, coupled with the consumption of highly palatable and energy-dense foods has led to increased prevalence and incidence rates of obesity and type-2 diabetes (T2DM) in recent decades. To date, more than 1 billion people worldwide have obesity [1] and 537 million people are affected by T2DM [2].

While the pathophysiology of obesity and T2DM is not fully elucidated, the recent research shows that, in humans, the brain may directly control whole body metabolic homeostasis [3,4,5,6,7]. However, the human brain remains the least accessible of all the organs. Thus, our potential in studying it in vivo relies predominantly on neuroimaging. Positron emission tomography (PET), magnetic resonance imaging (MRI), magnetic resonance spectroscopy (MRS), functional MRI (fMRI), and magnetoencephalography have been used to study brain metabolism in the context of metabolic diseases [8,9,10,11,12]. For instance, fMRI studies have demonstrated altered connectivity patterns in patients with T2DM and in people with obesity. On the one hand, the decreased connectivity in patients with T2DM is associated with a wide range of cognitive impairments [13]; on the other hand, the dysfunctional connectivity in people with obesity is suggestive of increased reward sensitivity and decreased interoceptive awareness [14]. However, the imaging modalities are very expensive investigations, with only few available specialized centers around the globe, thus limiting their use in large-scale studies.

Near-infrared spectroscopy (NIRS) or optical brain imaging is a method that was first described by Jöbsis in 1977 showing that there was a good transparency of biological materials in the near-infrared region of the light spectrum, and demonstrated that it was possible to measure changes in brain oxygenation in a cat using near-infared red light [15]. Since then, this method has been used to assess brain oxygenation in clinics in the context of general anesthesia; however, recently, it has been “re-discovered” for its potential in the context of brain metabolic studies as an alternative approach for studying neuronal activity.

Compared to the state-of-the art methods for studying the brain, NIRS has the advantage of not using ionizing radioactivity (PET), to be less expensive (PET and MRI), and to allow studies in conditions that are closer to free living. In the present narrative review, we describe the concept of NIRS in studying cortical activity in the context of diabetes, obesity, and binge eating disorder (BED, i.e., a disorder characterized by recurrent episodes of eating large quantities of food followed by a feeling of loss of control during the binge episode and experiencing guilt afterwards), conditions that all share metabolic disequilibrium that can directly affect neurovascular function and cognition, consequently. We conducted this overview in order to highlight the main findings of the previous studies and their associations with cognitive dysfunction or behavioral alterations, the possible limitations of the aforementioned studies, the future perspectives in this research line, and the potential future applications of NIRS for an improved understanding of brain pathophysiology in the context of metabolic disorders.

## 2. NIRS Concept—How It Works and Clinical Applications

**Figure 1:**
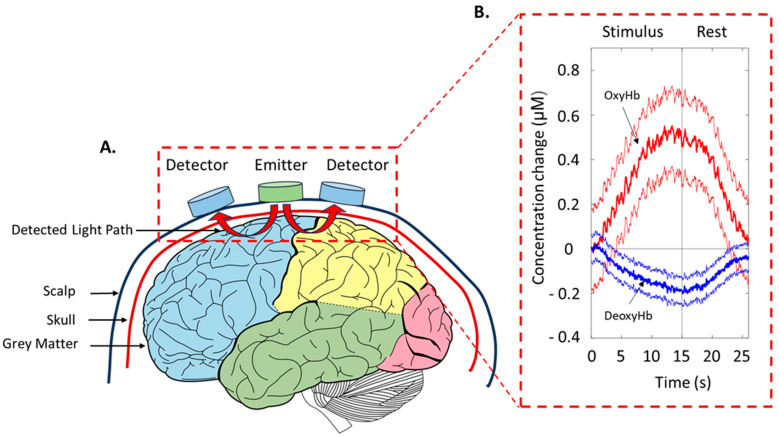
Emitter and detector arrangements on an adult human subject. (**A**) A two-channel emitter-detector pair is placed on the scalp. Arrows from the emitter to the detectors indicate the measured light path of each channel. (**B**) Example of concentration change (±standard deviation) of oxyhemoglobin (OxyHb) in red and deoxyhemoglobin (DeoxyHb) in blue during a stimulus and resting state. Personal data.

Functional near-infrared spectroscopy (fNIRS) is a non-invasive optical functional imaging system that is based on a near-infrared (NIR) optical window (700–900 nm), which is almost transparent to the skin, bone, and brain tissue [15]. A brain fNIRS signal is generated by shining NIR light (650–950 nm) into the head through the scalp and can reach the surface of the cerebral cortex. In its way, through the layers of the head, NIR light is subjected to “absorption” and “scattering”. Scattering leads to light attenuation and is much more frequent than absorption; therefore, placing a light detector at a certain distance from the NIR light allows us to register the backscattered light and measure the changes in light attenuation. Oxygenated (OxyHb) and deoxygenated (DeoxyHb) hemoglobin are chromophores that absorb near-infrared light, and most importantly, the absorption coefficients of OxyHb and DeoxyHb are different [16]. Therefore, the scattered-back light after absorption can provide information about the amount of chromophores in that region, which can be evaluated by the modified Beer–Lambert law (mBLL). From the difference between the intensity emitting and backscattered NIR light, the relative change in the oxygen concentration is assessed. In the hardware section of fNIR devices, the infrared light-emitter diode and detectors are generally placed 3–4 cm apart, as shown in Figure 1, oriented in a defined manner in an optode band, and it allows a 2 cm penetration depth. There are three different NIRS systems: continuous-wave NIRS is the oldest and most commonly used for its suitability and cost, despite its small light-penetration depth and the impossibility to separate light attenuation from absorption and scattering; time domain NIRS is less easily transportable, but has greater accuracy and spatial resolution, as light attenuation is measured by special cameras or single-photon counters based on their arrival time; and frequency domain NIRS uses a laser diode to emit light and it measures the attenuation, phase shift, and modulation depth of the light with respect to the system’s incident light [17].

When a brain area is active and involved in the execution of a certain task, the cerebral blood flow (CBF) increases to satisfy the metabolic demand of the brain. This increase in local blood flow in response to neuronal activation is called “functional hyperemia” and results from neurovascular coupling, mediated primarily by the vasoactive metabolites of arachidonic acid, nitric oxide, K+, and other mediators whose properties are partially still unknown [18]. The amount of oxygen that reaches the activated brain region is higher than the rate at which oxygen is consumed, leading to an increase in OxyHb and decrease in DeoxyHb. This is called “hemodynamic response” (HDR) and can be measured through fNIRS at multiple locations of the cerebral cortex. The HDR reaches a peak a few seconds after the stimulus onset and returns to its baseline with a certain delay (i.e., these can vary across different brain regions, task types and design, and participants’ age). The stimulus events are generally presented in block designs that consist of recurring blocks of tasks and rest periods; this configuration lets the HDR increase and return to the baseline level and has the highest signal-to-noise ratio, statistical power, and maximal time efficiency. By the early 1990s, fNIRS recordings demonstrated the capability of this technique to measure hemodynamic changes in the brain in response to functional activation tasks (e.g., flashing checkerboard at different contrast levels to study visual cortical activation or the Stroop test to study frontal, temporal, and anterior cingulate cortexes) both in adults and infants [19,20]. The development of more complex configurations made possible its use in cognitive experiments, allowing the study of functional brain activity and higher cognitive functions. In recent years, fNIRS found its application in functional neuroimaging techniques, which already included EEG/MEG, fMRI, and PET. The major advantage of fNIRS compared with EEG is that it is less susceptible to moving artefacts and offers a better spatial resolution, thus allowing for the localization of brain responses to specific cortical regions; however, EEG has a better temporal resolution (0.001 s) [19]. In addition, compared with fMRI, fNIRS has a better temporal resolution (0.02 s) and high temporal sampling rate (50 Hz), is silent, and can measure both Oxy- and DeoxyHb chromophores providing a more complete measure of the hemodynamic response; nevertheless, fMRI has a spatial resolution of 1 mm, which is better than fNIRS that has a spatial resolution of 1 cm [20]. Importantly, the comfort and absence of physical constraints on the participant allow for fNIRS’s use in a variety of settings, including infants, children, or the elderly, for long recording sessions, and resembles free-living conditions. As the optical components do not interfere with electromagnetic fields, fNIRS is ideal for multimodal imaging, for example, it can be combined with fMRI or EEG [21,22] to allow for the gathering of more complete information related to neurovascular coupling.

The fNIRS modality is being used in many areas of research, ranging from muscle physiology evaluations during exercise or pathologic conditions, such as metabolic myopathies and muscular dystrophies, chronic heart failure, and peripheral arterial disease in diabetes [21]. One of the most important and emerging clinical applications of fNIRS technology is aimed at the study of brain tissue and it has provided applications in several areas, including cerebral oximetry monitoring for adult patients undergoing cardiovascular surgery [22], neonatology to assess cerebral autoregulation in preterm infants, and the management of newborns with hypoxic ischemic encephalopathy. Moreover, fNIRS technology is also increasingly used in several neuroscience areas, including neurodegenerative diseases, such as Alzheimer’s disease [23,24], schizophrenia [25], addiction [26], dyslexia [27], attention-deficit/hyperactivity disorder [28,29], stroke rehabilitation [30], autism [31], depression [32], epilepsy [33], and migraine [34]. Based on the characteristics of the methodology underlying fNIRS and brain functional information provided, fNIRS does not contribute directly to diagnoses; rather, it is an assistive device that reads the functional activities of the brain and indirectly informs us regarding abnormalities in brain functionality. This implies that, in clinical practice, neurosurgeons or neurologists utilize the feature of this modality for anesthetic-depth monitoring to confirm that the patient is in a deeply sedated state. In some other mentioned applications, such as Alzheimer’s, schizophrenia, dyslexia, addiction, ADHD, epilepsy, depression, etc., fNIRS is used as a predictive modality that discriminates against the functional activity of hemodynamics with some behavioral tests.

## 3. The Complex Pathophysiology of Diabetes-Induced Alterations in the CNS

Diabetes, particularly when poorly controlled, can have significant effects on the structures and functions of the CNS leading to an increased risk for developing cognitive dysfunction and dementia [35]. Both type 1 diabetes (T1DM) and T2DM share the hyperglycemia phenotype that is known to affect the human brain and, in 2006, Mijnhout and colleagues proposed the term “diabetes-associated cognitive decline” [36]. The definition of diabetes-associated cognitive decline, however, has not yet been unanimously agreed upon [37] as the impact of diabetes on the CNS is multifaceted and can affect various aspects of neurological functions, including the alteration in structural integrity, networks connectivity and energy metabolism.

Cognitive alterations may differ between T1DM and T2DM, likely due to a relatively higher prevalence of other comorbidities in T2DM patients, such as obesity, hypertension, and lipid disorders. Indeed, subjects with T2DM presented greater neurodegeneration processes and a 26% increase in brain aging as compared to subjects without glucose tolerance abnormalities [38]. Subjects with T1DM presented lower-level performances on tests for attention, speed of information processing, and executive function [37] as compared to the control group. These alterations translate in mental slowing that is thought to be the fundamental cognitive domain impaired in T1DM, affecting both intelligence and psychomotor speed. More importantly, executive function, the components of which are working memory and response inhibition, is impaired in both adults and children with T1DM. In subjects with T2DM, the main altered domains are memory, information-processing speed, and executive functions [39,40], which are strictly dependent on brain cortical function. Exploring the impact of diabetes on cognitive function is extremely important for clinical reasons as cognitive dysfunction affects different behaviors that, in turn, impact on diabetes self-care, including reduced compliance in monitoring glucose, taking medication, following new instructions for diabetes management, and reducing the risk of hypoglycemia [41].

A key mechanism behind the impact of diabetes on CNS is the hyperglycemia-related damage to the blood vessels supplying the CNS, resulting in a condition known as cerebrovascular disease. Vascular damage can manifest as the thickening, narrowing, or occlusion of the blood vessels, ultimately leading to reduced blood flow, oxygen supply, neurovascular coupling disruption, brain energy metabolism alterations, and an increased risk of ischemic events in the CNS [42,43,44]. In recent years, various neuroimaging techniques have been employed to study the impact of diabetes on brain structure and function and have also been used to define the structural and functional correlates of cognitive dysfunction in diabetes providing insights into the mechanisms underlying the CNS complications of the disease [45]. In particular, the main brain structural abnormalities that have been associated with the deterioration of cognitive function are the atrophy of the cerebral gray and white matter that can be focal or generalized [38], white matter hyperintensities [46], (lacunar) infarcts [47], and microbleeds [48]. Cerebral blood flow (CBF) is responsible for the delivery of nutrients to the brain [49] and is correlated to brain activity; under normal conditions there is a coupling between metabolically active regions and CBF [50]. Studies have observed that reduced total cerebral perfusion was associated with impaired cognitive function [51]. Moreover, a recent systematic review and meta-analysis of arterial spin-labeling studies conducted on subjects with T2DM demonstrated decreased CBF in the frontal, occipital, and parietal lobes, which are involved in cognitive domains mainly altered in subjects with diabetes [52]. Moreover, the alteration in brain glucose metabolism was demonstrated in studies employing brain [^18^F]FDG-PET. Brain glucose hypermetabolism (during conditions of insulin clamp [53]) appears as an early trait that characterizes subjects with obesity [54] and subjects at an increased risk of obesity [55] or prediabetes [56]. However, diabetes is also associated with whole brain glucose hypometabolism in multiple brain regions [57]. Taken together, these data may suggest that brain hypermetabolism may serve as a transient compensatory reaction to the initial neurodegenerative insult; however, it is progressively replaced by hypometabolism, as tissue loss becomes more severe in the chronic situation. Definitively, an efficient and healthy cognitive function is based on the correct functioning of the neurovascular unit, which presupposes an integrity cerebral cortex structure, a flow of cerebral blood for oxygen and nutrients supply, including glucose, and a correct glucose uptake.

In addition to cognitive dysfunction, individuals with T2DM are at a greater risk of developing dementia, in particular vascular dementia and neurodegenerative diseases, such as Alzheimer’s and Parkinson’s disease [35]. Rouch et al. [58] showed in their study on 375 older ambulatory subjects with a mild cognitive impairment that the conversion to dementia was only associated with increased arterial stiffness and not with intima-media thickness, carotid plaques, or carotid artery diameter. Recent computational simulations using a detailed multicompartmental neurovascular model [59] provided insights into how vessel stiffness determines hemodynamic oscillatory peaks in neurovascular tissue. In addition to increasing oxygen availability at sites located away from small vessels [60], these vessel oscillations support waste clearance within the brain parenchyma, specifically convective bulk flow drainage along the basement membrane of capillaries and arterial walls [61]. Therefore, changes in the blood vessel pulsatility in T2DM can impair the convective bulk flow drainage that helps prevent the accumulation of neurotoxic waste proteins associated with neurodegenerative diseases. Moreover, small-vessel oscillatory dysfunction can reduce nutrient availability at sites located at a distance from small vessels [60], where reduced glucose availability in the CNS can directly trigger behavioral deficits by promoting amyloid-beta and tau neuropathology and synaptic dysfunction. A low-frequency Fahræus–Lindqvist-driven (not blood pressure-driven) oscillation in the small vessels is directly involved in the brain nutrient supply and can be considered a marker of vascular dysfunction.

## 4. fNIRS in Diabetes: A Promising Tool to Evaluate Central Nervous System Changes in Diabetes

fNIRS is a promising technique capable of exploring, albeit indirectly, the functioning of the neurovascular unit of the cerebral cortex, especially in the areas/lobes that underlie the main cognitive domains altered in diabetes. In a study conducted on T1DM patients, the fNIRS of the visual cortical areas was employed to explore the HDR that occurred as a reflex consequence to increased neuronal activity within the primary visual cortex in response to a standardized visual stimulation. Individuals with T1DM had a larger HDR compared to those with T2DM, and a linear relationship between HbA_1c_ level and HDR was observed, suggesting an autonomic nervous system dysfunction, either by the over-action of the sympathetic or under-action of the parasympathetic branches [62]. In a pilot study conducted on young adolescents with T1DM, the executive function was assessed through fNIRS during a Go/No-Go response-inhibition task. Individuals with T1DM had a similar performance task as compared to the healthy controls, but higher activations in the frontoparietal network, including the bilateral supramarginal gyri and bilateral rostrolateral prefrontal cortices. The activations in these regions were positively correlated with fewer parent-reported rule-breaking behaviors suggesting a link between this brain network and better self-control. These findings are consistent with a large fMRI study of children with T1DM based on different groups of participants [63]. Executive function is also impaired in older adults with T2DM, as it was demonstrated by the findings of greater gait variability and postural instability during walking under dual-tasking conditions, irrespective of the presence of diabetic polyneuropathy [64]. Although the impact of T2DM on CNS sensorimotor regions is understudied, there is evidence of the atrophy of the primary cortex (M1), secondary motor cortex (M2), and primary somatosensory cortex (S1), and also white matter projections between the sensorimotor cortices and subcortical structures are thought to be degenerated [65]. Van Harten et al. found a correlation between periventricular white matter hyperintensity in an MRI and poor motor speed that was also independently associated with the duration of T2DM [66]. With respect to these acknowledgements, fNIRS studies found a decline in OxyHb in the sensory and motor cortexes with simultaneous deficits in manual motor tasks in post-menopausal women with T2DM and in the prefrontal cortex (PFC) of diabetic older adults during attention-demanding locomotion tasks suggesting the role of PFC in the control of walking, notably under attention-demanding dual-task conditions [67]. These results may provide the evidence of a cortical contribution to motor dysfunction that is notoriously considered the result of peripheral nerve damage [68].

Similar to T1DM, subjects with T2DM are characterized by visuospatial dysfunctions, as demonstrated by a study exploring the impact of a pattern-reversal checkerboard stimulation on the primary visual cortex, which confirms an alteration in HDR as compared to the healthy controls [62]. These results obtained with fNIRS support the findings of our previous studies demonstrating in individuals with obesity and prediabetes an abnormal visual cortex plasticity measured by the effect of short-term monocular deprivation on binocular rivalry dynamics [69,70] and an fMRI study showing a reduction in neuronal activity in the lingual gyrus (visual cortex) that was correlated with poor performance on visuospatial tests [71].

Older T2DM individuals experience mild cognitive impairment, specifically in the domain of recall/working memory, and it may be exacerbated in older adult females, who are at the highest risk of cardiovascular decline due to diabetes. In an fNIRS study exploring OxyHb and DeoxyHb during memory-based tasks in a cross-sectional sample of postmenopausal women with T2DM, a deficit in working memory accuracy was associated with differences in OxyHb responses, altered PFC activity magnitudes, and increased functional cortical activity across the region of interests compared to the controls. These data indicate a shift of OxyHb in cortical activity patterns with memory deficits in postmenopausal T2DM, representing a novel diabetes-specific finding that is unlikely to be detected by fMRI [72]. This underscores the value of using non-MRI-based neuroimaging techniques to evaluate cortical hemodynamic function to detect early mild cognitive impairment. Recently, it was demonstrated that, in individuals with T2DM, deficits in working memory and reaction time might be improved by integrated yoga practice as compared to the control, and the improvements were associated with higher oxygenation in dorsolateral and ventrolateral PFC regions [73].

Elderly individuals with T2DM demonstrated a drop in vascular reactivity during Mini-Cog with a three-item recall test in the PFC, as compared to age-matched controls [74], suggesting that fNIRS captures cerebrovascular reactivity to cognitive load and it may provide a biomarker for cerebrovascular dysfunction in T2DM. Experimental studies have proven the neuroprotective effect of GLP-1 receptor agonists by ameliorating cognitive impairment in subjects with T2DM [75,76,77]. A recent study tried to clarify if this effect was due to a direct action on the central nervous system or the consequence of better metabolic control. Li et al. demonstrated that treatment with Liraglutide for 12 weeks improved cognitive function, as revealed by the increased brain activation assessed with fNIRS in cortex regions related to better cognitive performance, regardless of changes in blood pressure, glycemia, and body weight in patients with type-2 diabetes compared with regular hypoglycemic treatment [78]. These findings are congruent with the knowledge that GLP-1 and its receptors (GLP-1R) are also expressed in the brain, especially in the hippocampus (crucial for learning and memory), and that mice lacking GLP-1R present impaired associative contextual learning that can be reversed by hippocampal GLP-1R somatic cell gene transfer [79]. In conclusion, individuals affected by T1DM or T2DM have neural slowing, increased cortical atrophy, white matter lesions, and modified cerebral perfusion, which impair brain function differently, even if with some similarities. Certainly, further studies are needed to better understand the relationship between diabetes mellitus, cerebrovascular pathophysiology, and their implications on neurological function. The studies regarding the application of fNIRS studies on patients with diabetes are summarized in Table 1.

### Studies Using Brain NIRS to Study Inhibitory Control in Patients with Obesity or Binge Eating Disorder

In westernized societies, we live within an “obesogenic” environment where our senses (visual, or olfactory) are continuously exposed to palatable foods with a high caloric content. In such an environment, maintaining a healthy diet and avoiding the overconsumption of unhealthy foods requires cognitive control over our eating behavior. Previous fMRI studies have shown that frontal and prefrontal regions are activated in volitional appetite control, and this activation is blunted in patients with obesity [80]. These data suggest that dysfunctional frontal circuitry involved in inhibitory control may contribute to the wrong eating habits in patients with obesity. An excessive activation of the brain’s reward circuitry from palatable foods has also been described in patients with obesity [81].

Since the activity of the brain cortex and, in particular, the prefrontal cortex can be assessed with NIRS, studies employing NIRS have been used in studying the inhibitory control in patients with obesity and healthy lean controls. In this context, BED has also been studied, since subjects with BED episodically exhibit a loss of control of what they are consuming, leading to the consumption of a high number of calories. In both young and middle-aged overweight subjects, obesity, or BED, it has been shown that the activation of the PFC to food stimuli or the Stroop test is attenuated compared to healthy lean controls [82,83].

Veit et al. studied patients with BED and healthy controls at baseline and 3 months following a cognitive intervention program. The subjects were studied with NIRS during a food go/no-go task. In particular, subjects were shown healthy and un-healthy foods; in the first experiment, they were instructed to “go” for the healthy foods and “no-go” for the unhealthy foods, and in the second experiment, they were instructed to perform the opposite (i.e., “no-go” for healthy foods and “go” for unhealthy foods). Patients with BED had decreased right prefrontal cortex activation compared to the healthy subjects and, following impulsivity-focused cognitive treatment, they increased their right prefrontal activity during inhibition. Moreover, this increase in prefrontal activity was associated with a reduction in trait impulsivity [84].

Xu and colleagues performed a prospective weight loss study with a combination of exercise training and food restriction on 31 overweight or obese adolescents and young adults. The Stroop test was performed at baseline in conjunction with NIRS and following a 4-week fitness investigation. The authors reported a positive correlation between the hemodynamic interference due to the Stroop effect in the bilateral dorsolateral prefrontal cortex (DLPFC), left ventrolateral PFC (l-VLPFC), and left frontopolar area (l-FPA), and the amount of weight loss achieved [85]. The findings, which are in line with the previous fMRI studies [86], suggest that increased brain activation in the regions involved in executive function is associated with better weight loss outcomes.

Finally, Huang et al. studied the effect of age on brain activation during the Stroop test in 38 children, adolescents, and young adults with obesity aged 9 to 25 years old. They found that Stroop interference was not increased as a function of age [87]. This study’s results contrast with the findings of a previous study on children, adolescents, and young adults aged from 7 to 29 years old with a normal weight, where brain activation during the Stroop test was shown to be positively associated with age in the DLPFC [88]. Thus, as the authors discussed, these findings could be interpreted as evidence that, in the context of obesity, the neural mechanisms that are needed to suppress the Stroop effect are not as developed as in normal-weight individuals [87].

In conclusion, most NIRS studies assessed prefrontal cortex activation in response to inhibitory food or non-food stimuli, with subjects with obesity or BED having reduced pre-frontal activation in response to inhibitory food or non-food stimuli. Interestingly, following an intervention, such as cognitive treatment, prefrontal activation is restored. These findings are in line with recent intervention studies where non-invasive brain stimulation with transcranial direct-current stimulation (tDCS) over the right DLPFC induced a decrease in self-reported cravings, probably by strengthening inhibitory control [89], and tDCS targeted to bilaterally stimulate the PFC and insula decreased impulsivity and consequently BMI in patients with obesity [90]. The original fNIRS studies treating these themes are summarized in Table 2.

## 5. Future Applications—Adding Continuous Glucose Monitoring and Glucose Tolerance Tests to NIRS

Continuous glucose monitoring (CGM) allows for the assessment of intraday glycemic variability. On the other hand, the assessment of glycemic variability is known to be important for the study of brain function. In fact, it was demonstrated that both hyperglycemia and hypoglycemia can be associated with patient experiences of physical, affective, and cognitive symptoms, as well as cognitive-motor disruptions [91]. Of note, even mild or transient hypoglycemia can reduce the mental efficiency; although, the impact of this effect depends on the task the patient is dealing with [91]. In addition, both hypoglycemia and hyperglycemia have not only acute, but also chronic, effects on patients with T1DM and T2DM [91].

With regards to cognitive function, it has been demonstrated that there is a relationship between intraday glycemic variability (calculated by a common CGM metric named mean amplitude of glycemic excursions, MAGEs [92]) and cognitive performance, as assessed by cognitive tests, such as a composite score of executive and attention functioning and the mini mental status examination (MMSE) [93]. Interestingly, the association between MAGE and the impairment in cognitive function was independent from age, sex, body mass index, waist-to-hip ratio, drug intake, physical activity, mean arterial blood pressure, glycated hemoglobin, as well as the absolute glycemic values, both in fasting and post-prandial states [93]. It has also been hypothesized that glycemic variability can be responsible for cerebrovascular damage [94]. In fact, it has been shown that, in people with metabolic syndrome (although without T2DM), glycemic variability is associated with reduced cerebral vasomotor reactivity, and the association is independent from daily mean systolic and diastolic blood pressure levels [94]. Glycemic variability was also found to be associated with several stroke risk factors and with poorer short-term prognosis [95]. Furthermore, glycemic variability was found to be associated with cerebral metabolic distress (defined as lactate/pyruvate ratio >40), as well as hospital mortality, after subarachnoid hemorrhage [96]. On the other hand, a recent study emphasized that it was still partially unclear whether the association between glycemic variability and brain dysfunction following subarachnoid hemorrhage was due to direct deleterious effects induced by glucose dysregulation or if hyperglycemia was an epiphenomenon related to initial bleeding severity [97]. The study concluded that further research was needed to understand the most appropriate and informative timing for glucose monitoring and how to combine different glucose metrics with markers of brain injury [97]. The importance of selecting the most appropriate metrics for CGM data analysis for the study of brain function was also indicated in another recent study, which showed that, in T2DM patients, the glucose coefficient of variation (CV) and time below range (TBR) were not associated with any of the investigated aspects of cognitive function [98]. In contrast, some hyperglycemia metrics and the time in range (TIR) were associated with cognitive function, especially with executive function and working memory [98]. The reported findings indicate that there is certainly an association between glycemic variability and several aspects of brain function; however, there are still issues to be elucidated, including the identification of the most appropriate CGM metrics and brain function markers to shed further light onto such an association. In fact, further studies, even of a longitudinal type, are ongoing at present [99].

It also has to be noted that some studies also investigated the possible molecular mechanisms determining the association between glycemic variability and altered brain function. In fact, one study investigated the possible mechanistic basis for diabetes-induced cerebrovascular damage, due to both hyperglycemia and high glycemic fluctuations [100]. It was shown that carbonyl stress, indicated by the formation of occluding-methylglyoxal carbonyls, was a possible mechanism of endothelial barrier dysfunction [100]. The study also demonstrated that hyperglycemia compromised the elimination of methylglyoxal by the glyoxalase pathway and, hence, increased the glycating potential of methylglyoxal, thus exacerbating the dysfunction of the cerebral microvasculature [100]. Another study evaluated the responses of neuronal cells to different glycemic exposures and investigated the role of mitochondrial uncoupling proteins (UCPs) in regulating such responses, with a focus on one such protein (UPC2) [101]. Indeed, UCPs are mitochondrial anion carriers with a crucial contribution to regulating mitochondrial homeostasis, and have a demonstrated role in physiological and pathological adaptations of the brain [101]. The study findings suggested that UCP2 was at the core of neuronal cell protection or adaptation against the effects mediated by glycemic variability, and that other isoforms of neuronal UCPs can be upregulated to compensate for the possible inhibition of UCP2 activity [101].

The studies summarized above provide evidence of the clear association between glycemic variability and brain function impairment, while, at the same time, indicating that further research is still necessary to clarify some issues, as previously reported. In our opinion, one of the possible future directions for such new studies is the integration of CGM data with modern neuroimaging techniques, such as fNIRS. Indeed, among the previous studies conducted on glycemic variability and brain function, those that specifically exploit neuroimaging data are rare. One study investigating the association between glycemic variability at multiple time scales and brain volumes exploited MRI [71]. The anatomical MRI data were segmented to calculate regional gray and white matter and cerebrospinal fluid volumes in the main anatomical lobes and their sub-regions [71]. MRI was also used to assess the association between glycemic variability and brain damage in hypoglycemia neonates [102]. Another study, assessing the association between glycemic variability and the presence of cerebral lacunes (sign of lacunar infarction potentially leading to dementia or disability), retrospectively analyzed MRI data to identify such lacunes [103]. FMRI was used in a study investigating the associations between glycemic variability, hypothalamic function, and diet during the first 18 months of life [104]. With regard to studies integrating glycemic variability data with NIRS data, we did not identify any study, thus indicating that the combination of CGM and NIRS would definitely be a new strategy.

In addition to the CGM data for the assessment of glycemic variability, in our opinion, NIRS imaging may also be conveniently integrated with glucometabolic information derived by a glucose (or meal) tolerance test, with special interest in the traditional oral glucose tolerance test (OGTT). In fact, some previous studies, though still few, performed OGTT while investigating some aspects of brain function. Specifically, most of these studies focused on the investigation of the brain-derived neurotrophic factor (BDNF), which is a member of the neurotrophin family and plays an important role in neural protection, synaptic activity, and endothelial survival. One study investigated whether circulating BDNF levels change during an OGTT and found that plasma levels of BDNF were sensitive to acute changes in both OGTT glucose and insulin levels [105]. In another study, it was shown that, after an oral glucose challenge, a lower serum BDNF response was associated with higher central pulse pressure [106]. Another study by the same research group of study [106] showed that lower serum BDNF levels during the OGTT were associated with high cardiovascular risk [107]. Another study in the field addressed a different topic, since it focused on multiple sclerosis (MS), which is a chronic neurologic condition predominantly affecting young people and, in fact, one of the major causes of disability in this population [108]. That study investigated the association of glucose metabolism, as assessed by an OGTT, with the level of MS progression and related degree of disability, and a strong association was found between impaired glucose metabolism and disability [108].

In summary, based on the reported previous studies, we claimed that the integration of fNIRS, CGM, and OGTT data would likely provide invaluable information and increase the scientific scenarios concerning the study of brain function. In fact, such a “triple integration” would be an extremely innovative approach.

## 6. Conclusions

In conclusion, fNIRS is a relatively cheap neuroimaging tool that allows for the study of cortical networks in conditions resembling free-living conditions. Since it does not employ the use of radiation, experiments can be performed in different occasions. Several alterations have already been identified in both patients with T1DM, T2DM, and patients with obesity. Further applications of fNIRS, for instance, in concomitance of CGM, or before and after interventions (weight loss, drug treatment, bariatric surgery, exercise), may improve our understanding of the interplay between systemic metabolism and cortical networks.

## Figures and Tables

**Table 1:** Summary of original articles using NIRS in patients with diabetes.

Reference	Subjects	Age	NIRS System	Experimental Procedure	Results	Limitations
Aitchinson et al. [54]	15 pts (8 males) with diabetes (5 T1DM, 10 T2DM); 15 (7 males) HC	Diabetes: 47 ± 19 years old HC: 46 ± 23 years old	2-channel CW fNIRS on occipital cortex (V1)	7 cycles of 30 s of pattern-reversal checkerboard stimulation and 30 s of gray screen	Greater HDR in diabetic pts compared to controls; greater HDR in T1DM pts compared to T2DM pts	-Little brain surface covered by fNIRS type-T1DM and T2DM merged in the same group when performing between-group comparisons -Small sample size
Gorniak et al. [64]	21 post-menopausal women with T2DM; 21 post-menopausal controls	T2DM: 65 ± 6 years old HC: 67 years old	28-channel CW fNIRS on prefrontal, motor, and sensory cortices	fNIRS measurements during N-back task and N-back task + motor task	Decreased accuracy and increased reaction times increase in OxyHb concentration in the diabetes group compared to HC	Small sample size
Li et al. [74]	24 T2DM pts (14 males) in liraglutide group; 23 T2DM pts (9 males) in control group	Intervention group: 55 ± 12 years old CT: 60 ± 7 years old	20-channel CW fNIRS on the prefrontal cortex	fNIRS measurement during verbal fluency task and neuropsychological assessment before and after 12 weeks of treatment in liraglutide group and after 12 weeks with no treatment in control group	Increase in OxyHb concentration in DLPF and OFC and higher MMSE, total learning, and animal naming test scores in liraglutide group compared to control group	-fNIRS measurement conducted only during verbal fluency test and not while performing all the other tests -Small sample size
Mazaika et al. [55]	19 T1DM pts (9 males); 18 HC (10 males)	T1DM: 12 ± 3 years old HC: 11 ± 3 years old	48-channel CW fNIRS on the lateral prefrontal cortex, superior temporal gyrus, postcentral gyrus, and supramarginal gyrus	fNIRS measurement during Go/No-Go task	No significant differences in accuracy and response time; higher frontoparietal activations in diabetes group compared to HC	-Small sample size -Young age could have prevented significant differences in behavioral data due to high brain plasticity
Holtzer et al. [59]	43 T2DM pts (37 with oral hypoglycemic therapy and 6 with insulin therapy; 272 HC; 56.5% females of all subjects	All subjects: 77 ± 7 years old	CW fNIRS with 4 sources on the prefrontal cortex	fNIRS measurement during normal-walk task, cognitive interference task, and walk-while-talking task	Higher OxyHb concentration and lower performance during cognitive interference in diabetes group compared to control group; lower OxyHb concentration during walk-while-talking task in diabetes group compared to HC	Imbalanced sample size of diabetic and control groups and of diabetic subgroups
Kaligal et al. [65]	25 T2DM pts in yoga treatment group; 25 T2DM pts in no treatment group; 26 males of all subjects	62 ± 6 years old	24-channel CW fNIRS on the prefrontal cortex	fNIRS measurement during 3 blocks of 20 trials of N-back task alternated with 20 s of rest at baseline and after 6 and 12 weeks of yoga in the yoga group and after 6 and 12 weeks with no treatment in the control group	Improved accuracy and reaction times associated with increased OxyHb concentration in the yoga group compared to baseline	Small sample size
Zhao et al. [70]	19 elderly individuals with T2DM and 38 HC	60 years old	CW fNIRS with 4 sources and 2 detectors on the prefrontal cortex	fNIRS measurement during Mini-Cog three-item recall test	Decrease in power in the 0.01–0.02 Hz frequency band more pronounced in the diabetes group compared to control group	Imbalanced sample size of groups

DLPFC: dorsolateral prefrontal cortex; CW: continuous wave; fNIRS: functional near-infrared spectroscopy; HC: healthy controls; HDR: hemodynamic response; OFC: orbitofrontal cortex; pts: patients; PwDM: post-menopausal women with T2DM; RCT: randomized-controlled trial; T1DM: type-1 diabetes; T2DM: type-2 diabetes.

**Table 2:** Summary of original articles using NIRS in patients with obesity or BED compared to lean controls.

Reference	Subjects	Age	NIRS System	Experimental Procedure	Results	Limitations
Rösch et al. [81]	15 pts (9 females) with OB; 13 pts (11 females) with OB+BED; 12 HC (8 females)	OB group: 50 ± 18 years old; OB+BED: 43 ± 13 years old; HC: 56 ± 19 years old	28-channel CW NIRS on the prefrontal cortex	fNIRS measurement during passive viewing of 5 blocks of 12 stimuli each of appetitive pictures of food and Go/No-Go task with 6 blocks of 12 selected food pictures	Decreased response of prefrontal cortex in OB and OB+BED groups compared to control group and in BED group compared to OB group in both tasks	-Small sample size -No behavioral data of Go/No-Go task to be compared between groups and to be correlated with fNIRS data
Deng et al. [82]	15 pts (9 males) with OB; 17 (8 males) subjects with overweight	OB: 20 ± 2 years old; overweight: 20 ± 2 years old	20-channel CW NIRS on the prefrontal cortex	fNIRS measurement during 48 trials (24 congruent and 24 incongruent) of Stroop task	Greater interference effect in OB group compared to overweight group; decreased HDR in subjects with elevated waist circumference and BMI	-Small sample size -No control group with normal-weight individuals
Huang et al. [86]	38 pts (24 males) with OB	Males: 17 ± 5 years old; females: 15 *±* 5 years old	20-channel CW fNIRS on the prefrontal cortex	fNIRS measurement during 48 trials (24 congruent and 24 incongruent) of Stroop task	No significant association between age and behavioral/hemodynamic interference effect	No control group with normal-weight individuals
Veit et al. [83]	24 pts (20 females) with BED: 14 of them allocated to treatment group and 10 to no treatment group; 12 HC (7 females)	BED: 39 ± 12 years old; HC: 43 ± 13 years old	12-channel CW NIRS on the prefrontal cortex	fNIRS measurement during a 28 min Go/No-Go task with 12 blocks of healthy and unhealthy food before and after 8 weekly sessions of cognitive treatment and after 3 months from treatment	Weaker activation of the prefrontal cortex during response inhibition in BED group compared to healthy subjects; increased activation following treatment in BED group with treatment compared to BED group with no treatment after 3 mo	Imbalanced number of males and females
Xu et al. [84]	31 subjects (12 females) with OB or overweight	18 ± 3 years old	20-channel CW fNIRS on the prefrontal cortex	fNIRS measurement during 48 trials (24 congruent and 24 incongruent) of Stroop task	Reduced reaction times and increased HDR during interference in subjects that lost more weight	No control group with normal-weight individuals
Rhee et al. [90]	14 females with OB; 14 females without OB; 11 males with OB; 14 males without OB	Males without OB: 75 ± 6 years old; males with OB: 73 ± 7 years old; females without OB: 72 ± 4 years old; females with OB: 72 ± 6 years old	26-channel CW NIRS on the prefrontal, motor, and sensory areas	fNIRS measurement during 3 min of rest and during 3 trials of motor fatigue test	Increased variability in functional connectivity during rest in the group with OB compared to the group without OB; increased variability in functional connectivity during task in group without OB compared to group with OB; increased connectivity between all nodes in males compared to females; greater fatigue-related response of Oxy-Hb in the contralateral sensory area in group without OB compared to group with OB; lower coefficient of variability in motor performance in group with OB compared to group without OB	Small sample size

BED: binge-eating disorder; BMI: body mass index; CW: continuous wave; DLPCF: dorsolateral prefrontal cortex; HDR: hemodynamic response; HC: heathy controls; OB: obesity.

## References

[1] World Obesity Prevalence of Obesity. https://www.worldobesity.org/about/about-obesity/prevalence-of-obesity.

[2] Saeedi P., Petersohn I., Salpea P., Malanda B., Karuranga S., Unwin N., Colagiuri S., Guariguata L., Motala A.A., Ogurtsova K. (2019). Global and regional diabetes prevalence estimates for 2019 and projections for 2030 and 2045: Results from the International Diabetes Federation Diabetes Atlas, 9(th) edition. Diabetes Res. Clin. Pract..

[3] Rebelos E., Immonen H., Bucci M., Hannukainen J.C., Nummenmaa L., Honka M.-J., Soinio M., Salminen P., Ferrannini E., Iozzo P. (2019). Brain glucose uptake is associated with endogenous glucose production in obese patients before and after bariatric surgery and predicts metabolic outcome at follow-up. Diabetes Obes. Metab..

[4] Rebelos E., Mari A., Bucci M., Honka M.-J., Hannukainen J.C., Virtanen K.A., Hirvonen J., Nummenmaa L., Heni M., Iozzo P. (2020). Brain substrate metabolism and ß-cell function in humans: A positron emission tomography study. Endocrinol. Diabetes Metab..

[5] Rebelos E., Hirvonen J., Bucci M., Pekkarinen L., Nyman M., Hannukainen J.C., Iozzo P., Salminen P., Nummenmaa L., Ferrannini E. (2020). Brain free fatty acid uptake is elevated in morbid obesity, and is irreversible 6 months after bariatric surgery: A positron emission tomography study. Diabetes Obes. Metab..

[6] Heni M., Wagner R., Kullmann S., Gancheva S., Roden M., Peter A., Stefan A., Preissl H., Häring H.-U., Fritsche A. (2017). Hypothalamic and Striatal Insulin Action Suppresses Endogenous Glucose Production and May Stimulate Glucose Uptake During Hyperinsulinemia in Lean but Not in Overweight Men. Diabetes.

[7] Heni M., Wagner R., Willmann C., Jaghutriz B.A., Vosseler A., Kübler C., Hund V., Scheffler K., Peter A., Häring H.-U. (2020). Insulin Action in the Hypothalamus Increases Second-Phase Insulin Secretion in Humans. Neuroendocrinology.

[8] Rebelos E., Bucci M., Karjalainen T., Oikonen V., Bertoldo A., Hannukainen J.C., Virtanen K.A., Latva-Rasku A., Hirvonen J., Heinonen I. (2021). Insulin resistance is associated with enhanced brain glucose uptake during euglycemic hyperinsulinemia: A large-scale PET cohort. Diabetes Care.

[9] Rebelos E., Nummenmaa L., Dadson P., Latva-Rasku A., Nuutila P. (2020). Brain insulin sensitivity is linked to body fat distribution-the positron emission tomography perspective. Eur. J. Nucl. Med. Mol. Imaging.

[10] Dardano A., Aghakhanyan G., Moretto C., Ciccarone A., Bellini R., Sancho Bornez V., Ceccarini G., Santini F., Volterrani V., Del Prato S. (2022). Brain effect of bariatric surgery in people with obesity. Int. J. Obes..

[11] Kullmann S., Valenta V., Wagner R., Tschritter O., Machann J., Häring H.-U., Preissl H., Fritsche A., Heni M. (2020). Brain insulin sensitivity is linked to adiposity and body fat distribution. Nat. Commun..

[12] Tschritter O., Preissl H., Hennige A.M., Stumvoll M., Porubska K., Frost R., Marx H., Klösel B., Lutzenberger W., Birbaumer N. (2006). The cerebrocortical response to hyperinsulinemia is reduced in overweight humans: A magnetoencephalographic study. Proc. Natl. Acad. Sci. USA.

[13] Lei H., Hu R., Luo G., Yang T., Shen H., Deng H., Chen C., Zhao H., Liu J. (2021). Altered Structural and Functional MRI Connectivity in Type 2 Diabetes Mellitus Related Cognitive Impairment: A Review. Front. Hum. Neurosci..

[14] Parsons N., Steward T., Clohesy R., Almgren H., Duehlmeyer L. (2022). A systematic review of resting-state functional connectivity in obesity: Refining current neurobiological frameworks and methodological considerations moving forward. Rev. Endocr. Metab. Disord..

[15] Jöbsis F.F. (1977). Noninvasive, infrared monitoring of cerebral and myocardial oxygen sufficiency and circulatory parameters. Science.

[16] Pinti P., Tachtsidis I., Hamilton A., Hirsch J., Aichelburg C., Gilbert S., Burgess P.-W. (2020). The present and future use of functional near-infrared spectroscopy (fNIRS) for cognitive neuroscience. Ann. N. Y. Acad. Sci..

[17] Almajidy R.K., Mankodiya K., Abtahi M., Hofmann U.G. (2020). A Newcomer’s Guide to Functional Near Infrared Spectroscopy Experiments. IEEE Rev. Biomed. Eng..

[18] Nippert A.R., Biesecker K.R., Newman E.A. (2018). Mechanisms Mediating Functional Hyperemia in the Brain. Neurosci. A Rev. J. Bringing Neurobiol. Neurol. Psychiatry.

[19] Ferrari M., Quaresima V. (2012). A brief review on the history of human functional near-infrared spectroscopy (fNIRS) development and fields of application. Neuroimage.

[20] Laguë-Beauvais M., Brunet J., Gagnon L., Lesage F., Bherer L. (2013). A fNIRS investigation of switching and inhibition during the modified Stroop task in younger and older adults. Neuroimage.

[21] Grassi B., Quaresima V. (2016). Near-infrared spectroscopy and skeletal muscle oxidative function in vivo in health and disease: A review from an exercise physiology perspective. J. Biomed. Opt..

[22] Hogue C.W., Levine A., Hudson A., Lewis C. (2021). Clinical Applications of Near-infrared Spectroscopy Monitoring in Cardiovascular Surgery. Anesthesiology.

[23] Arai H., Takano M., Miyakawa K., Ota T., Takahashi T., Asaka H., Kawaguchi T. (2006). A quantitative near-infrared spectroscopy study: A decrease in cerebral hemoglobin oxygenation in Alzheimer’s disease and mild cognitive impairment. Brain Cogn..

[24] Zeller J.B.M., Herrmann M.J., Ehlis A.-C., Polak T., Fallgatter A.J. (2010). Altered parietal brain oxygenation in Alzheimer’s disease as assessed with near-infrared spectroscopy. Am. J. Geriatr. Psychiatry Off. J. Am. Assoc. Geriatr. Psychiatry.

[25] Koike S., Takizawa R., Nishimura Y., Takano Y., Takayanagi Y., Kinou M., Araki T., Harima H., Fukuda M., Okazaki Y. (2011). Different hemodynamic response patterns in the prefrontal cortical sub-regions according to the clinical stages of psychosis. Schizophr. Res..

[26] Bunce S.C., Izzetoglu K., Izzetoglu M., Ayaz H., Pourrezaei K., Onaral B., Zhang H., Hussain A., Liu D., Wang Z. (2012). Treatment Status Predicts Differential Prefrontal Cortical Responses to Alcohol and Natural Reinforcer Cues among Alcohol Dependent Individuals. Advances in Brain Inspired Cognitive Systems.

[27] Sela I., Horowitz–Kraus T., Izzetoglu M., Shewokis P.A., Izzetoglu K., Onaral B., Breznitz Z., Schmorrow D.D., Fidopiastis C.M. (2011). Brain Activity of Young and Adult Hebrew Speakers during Lexical Decision Task: fNIR Application to Language. Foundations of Augmented Cognition. Directing the Future of Adaptive Systems.

[28] Blume F., Hudak J., Dresler T., Ehlis A.-C., Kühnhausen J., Renner T.J., Gawrilow C. (2017). NIRS-based neurofeedback training in a virtual reality classroom for children with attention-deficit/hyperactivity disorder: Study protocol for a randomized controlled trial. Trials.

[29] Monden Y., Dan H., Nagashima M., Dan I., Kyutoku Y., Okamoto M., Yamagata T., Momoi M.Y., Watanabe E. (2012). Clinically-oriented monitoring of acute effects of methylphenidate on cerebral hemodynamics in ADHD children using fNIRS. Clin. Neurophysiol. Off. J. Int. Fed. Clin. Neurophysiol..

[30] Nakamura S., Sakatani K., Kano T., Hoshino T., Fujiwara N., Murata Y., Katayama Y. (2010). Effects of revascularisation on evoked cerebral blood oxygenation responses in stroke patients. Adv. Exp. Med. Biol..

[31] Li Y., Yu D. (2018). Variations of the Functional Brain Network Efficiency in a Young Clinical Sample within the Autism Spectrum: A fNIRS Investigation. Front. Physiol..

[32] Nishizawa Y., Kanazawa T., Kawabata Y., Matsubara T., Maruyama S., Kawano M., Kinoshita S., Koh J., Matsuo K., Yoneda H. (2019). fNIRS Assessment during an Emotional Stroop Task among Patients with Depression: Replication and Extension. Psychiatry Investig..

[33] Machado A., Lina J.M., Tremblay J., Lassonde M., Nguyen D.K., Lesage F., Grova C. (2011). Detection of hemodynamic responses to epileptic activity using simultaneous Electro-EncephaloGraphy (EEG)/Near Infra Red Spectroscopy (NIRS) acquisitions. Neuroimage.

[34] Watanabe Y., Tanaka H., Dan I., Sakurai K., Kimoto K., Takashima R., Hirata K. (2011). Monitoring cortical hemodynamic changes after sumatriptan injection during migraine attack by near-infrared spectroscopy. Neurosci. Res..

[35] Biessels G.J., Staekenborg S., Brunner E., Brayne C., Scheltens P. (2006). Risk of dementia in diabetes mellitus: A systematic review. Lancet Neurol..

[36] Mijnhout G.S., Scheltens P., Diamant M., Biessels G.J., Wessels A.M., Simsek S., Snoek F.J., Heine R.J. (2006). Diabetic encephalopathy: A concept in need of a definition. Diabetologia.

[37] Moheet A., Mangia S., Seaquist E.R. (2015). Impact of diabetes on cognitive function and brain structure. Ann. N. Y. Acad. Sci..

[38] Antal B., McMahon L.P., Sultan S.F., Lithen A., Wexler D.J., Dickerson B., Ratai E.-V., Mujica-Parodi L.R. (2022). Type 2 diabetes mellitus accelerates brain aging and cognitive decline: Complementary findings from UK Biobank and meta-analyses. eLife.

[39] Brands A.M.A., Van den Berg E., Manschot S.M., Biessels G.J., Kappelle L.J., De Haan E.H.F., Kessels R.P. (2007). A detailed profile of cognitive dysfunction and its relation to psychological distress in patients with type 2 diabetes mellitus. J. Int. Neuropsychol. Soc..

[40] Reijmer Y.D., van den Berg E., Ruis C., Kappelle L.J., Biessels G.J. (2010). Cognitive dysfunction in patients with type 2 diabetes. Diabetes Metab. Res. Rev..

[41] Munshi M.N. (2017). Cognitive Dysfunction in Older Adults With Diabetes: What a Clinician Needs to Know. Diabetes Care.

[42] Hayden M.R. (2019). Type 2 Diabetes Mellitus Increases The Risk of Late-Onset Alzheimer’s Disease: Ultrastructural Remodeling of the Neurovascular Unit and Diabetic Gliopathy. Brain Sci..

[43] McCrimmon R.J., Ryan C.M., Frier B.M. (2012). Diabetes and cognitive dysfunction. Lancet.

[44] Rebelos E., Daniele G., Campi B., Saba A., Koskensalo K., Ihalainen J., Saukko E., Nuutila P., Backes W.H., Jansen J.F.A. (2022). Circulating N-Acetylaspartate does not track brain NAA concentrations, cognitive function or features of small vessel disease in humans. Sci. Rep..

[45] Cheah Y.-S., Amiel S.A. (2012). Metabolic neuroimaging of the brain in diabetes mellitus and hypoglycaemia. Nat. Rev. Endocrinol..

[46] De Groot J.C., De Leeuw F.-E., Oudkerk M., Van Gijn J., Hofman A., Jolles J., Breteler M.M.B. (2002). Periventricular cerebral white matter lesions predict rate of cognitive decline. Ann. Neurol..

[47] Vermeer S.E., Longstreth W.T.J., Koudstaal P.J. (2007). Silent brain infarcts: A systematic review. Lancet Neurol..

[48] Greenberg S.M., Vernooij M.W., Cordonnier C., Viswanathan A., Al-Shahi Salman R., Warach S., Launer L.L., Van Buchem A.A., Breteler M.M.B., Microbleed Study Group (2009). Cerebral microbleeds: A guide to detection and interpretation. Lancet Neurol..

[49] Fantini S., Sassaroli A., Tgavalekos K.T., Kornbluth J. (2016). Cerebral blood flow and autoregulation: Current measurement techniques and prospects for noninvasive optical methods. Neurophotonics.

[50] Hoge R.D., Atkinson J., Gill B., Crelier G.R., Marrett S., Pike G.B. (1999). Linear coupling between cerebral blood flow and oxygen consumption in activated human cortex. Proc. Natl. Acad. Sci. USA.

[51] Tiehuis A.M., Vincken K.L., van den Berg E., Hendrikse J., Manschot S.M., Mali W.P.T.M., Kappelle L.J., Biessels G.J. (2008). Cerebral perfusion in relation to cognitive function and type 2 diabetes. Diabetologia.

[52] Liu J., Yang X., Li Y., Xu H., Ren J., Zhou P. (2022). Cerebral Blood Flow Alterations in Type 2 Diabetes Mellitus: A Systematic Review and Meta-Analysis of Arterial Spin Labeling Studies. Front. Aging Neurosci..

[53] Rebelos E., Rinne J.O., Nuutila P., Ekblad L.L. (2021). Brain Glucose Metabolism in Health, Obesity, and Cognitive Decline-Does Insulin Have Anything to Do with It? A Narrative Review. J. Clin. Med..

[54] Tuulari J.J., Karlsson H.K., Hirvonen J., Hannukainen J.C., Bucci M., Helmiö M., Ovaska J., Soinio M., Salminen P., Savisto N. (2013). Weight loss after bariatric surgery reverses insulin-induced increases in brain glucose metabolism of the morbidly obese. Diabetes.

[55] Pekkarinen L., Kantonen T., Rebelos E., Latva-Rasku A., Dadson P., Karjalainen T., Kalliokoski K., Laitinen K., Houttu N., Kirjavainen A.K. (2022). Obesity risk is associated with brain glucose uptake and insulin resistance. Eur. J. Endocrinol..

[56] Hirvonen J., Virtanen K.A., Nummenmaa L., Hannukainen J.C., Honka M.-J., Bucci M., Nesterov S.V., Parkkola R., Rinne J., Iozzo P. (2011). Effects of insulin on brain glucose metabolism in impaired glucose tolerance. Diabetes.

[57] Iozzo P., Guzzardi M.A. (2019). Imaging of brain glucose uptake by PET in obesity and cognitive dysfunction: Life-course perspective. Endocr. Connect..

[58] Rouch L., Cestac P., Sallerin B., Andrieu S., Bailly H., Beunardeau M., Cohen A., Dubail D., Hernandorena I., Seux M.-L. (2018). Pulse Wave Velocity Is Associated With Greater Risk of Dementia in Mild Cognitive Impairment Patients. Hypertension.

[59] Arora Y., Walia P., Hayashibe M., Muthalib M., Chowdhury S.R., Perrey S., Dutta A. (2021). Grey-box modeling and hypothesis testing of functional near-infrared spectroscopy-based cerebrovascular reactivity to anodal high-definition tDCS in healthy humans. PLoS Comput. Biol..

[60] Geddes J.B., Carr R.T., Wu F., Lao Y., Maher M. (2010). Blood flow in microvascular networks: A study in nonlinear biology. Chaos.

[61] Kaur J., Fahmy L.M., Davoodi-Bojd E., Zhang L., Ding G., Hu J., Zhang Z., Chopp M., Jiang Q. (2021). Waste Clearance in the Brain. Front. Neuroanat..

[62] Aitchison R.T., Ward L., Kennedy G.J., Shu X., Mansfield D.C., Shahani U. (2018). Measuring visual cortical oxygenation in diabetes using functional near-infrared spectroscopy. Acta Diabetol..

[63] Mazaika P.K., Marzelli M., Tong G., Foland-Ross L.C., Buckingham B.A., Aye T., Reiss A.L. (2020). Functional near-infrared spectroscopy detects increased activation of the brain frontal-parietal network in youth with type 1 diabetes. Pediatr. Diabetes.

[64] Roman de Mettelinge T., Delbaere K., Calders P., Gysel T., Van Den Noortgate N., Cambier D. (2013). The impact of peripheral neuropathy and cognitive decrements on gait in older adults with type 2 diabetes mellitus. Arch. Phys. Med. Rehabil..

[65] Ferris J.K., Inglis J.T., Madden K.M., Boyd L.A. (2020). Brain and Body: A Review of Central Nervous System Contributions to Movement Impairments in Diabetes. Diabetes.

[66] van Harten B., Oosterman J., Muslimovic D., van Loon B.-J.P., Scheltens P., Weinstein H.C. (2007). Cognitive impairment and MRI correlates in the elderly patients with type 2 diabetes mellitus. Age Ageing.

[67] Holtzer R., George C.J., Izzetoglu M., Wang C. (2018). The effect of diabetes on prefrontal cortex activation patterns during active walking in older adults. Brain Cogn..

[68] Gorniak S.L., Wagner V.E., Vaughn K., Perry J., Cox L.G., Hernandez A.E., Pollonini L. (2020). Functional neuroimaging of sensorimotor cortices in postmenopausal women with type II diabetes. Neurophotonics.

[69] Lunghi C., Daniele G., Binda P., Dardano A., Ceccarini G., Santini F., Del Prato S., Morrone M.C. (2019). Altered Visual Plasticity in Morbidly Obese Subjects. iScience.

[70] Daniele G., Lunghi C., Dardano A., Binda P., Ceccarini G., Santini F., Giusti L., Ciccarone A., Bellini R., Moretto C. (2021). Bariatric surgery restores visual cortical plasticity in nondiabetic subjects with obesity. Int. J. Obes..

[71] Cui X., Abduljalil A., Manor B.D., Peng C.-K., Novak V. (2014). Multi-scale glycemic variability: A link to gray matter atrophy and cognitive decline in type 2 diabetes. PLoS ONE.

[72] Gorniak S.L., Wagner V.E., Vaughn K., Perry J., Cox L.G., Hibino H., Montero-Hernandez S.A., Hernandez A.E., Pollonini L. (2023). Functional near infrared spectroscopy detects cortical activation changes concurrent with memory loss in postmenopausal women with Type II Diabetes. Exp. Brain Res..

[73] Kaligal C., Kanthi A., Vidyashree M., Krishna D., Raghuram N., Hongasandra Ramarao N., Deepeshwar S. (2023). Prefrontal oxygenation and working memory in patients with type 2 diabetes mellitus following integrated yoga: A randomized controlled trial. Acta Diabetol..

[74] Zhao F., Tomita M.R., Dutta A. (2022). Functional near-infrared spectroscopy of prefrontal cortex during memory encoding and recall in elderly with type 2 diabetes mellitus. Annu. Int. Conf. IEEE Eng. Med. Biol. Soc..

[75] Cukierman-Yaffe T., Gerstein H.C., Colhoun H.M., Diaz R., García-Pérez L.-E., Lakshmanan M., Bethel A., Xavier D., Probstfield J., Riddle M.C. (2020). Effect of dulaglutide on cognitive impairment in type 2 diabetes: An exploratory analysis of the REWIND trial. Lancet Neurol..

[76] Vadini F., Simeone P.G., Boccatonda A., Guagnano M.T., Liani R., Tripaldi R., Di Castelnuovo A., Cipollone F., Consoli A., Santilli F. (2020). Liraglutide improves memory in obese patients with prediabetes or early type 2 diabetes: A randomized, controlled study. Int. J. Obes..

[77] Zhang Z., Zhang B., Wang X., Zhang X., Yang Q.X., Qing Z., Zhang W., Zhu D., Bi Y. (2019). Olfactory Dysfunction Mediates Adiposity in Cognitive Impairment of Type 2 Diabetes: Insights From Clinical and Functional Neuroimaging Studies. Diabetes Care.

[78] Li Q., Jia M., Yan Z., Li Q., Sun F., He C., Li Y., Zhou X., Zhang X., Liu X. (2021). Activation of Glucagon-Like Peptide-1 Receptor Ameliorates Cognitive Decline in Type 2 Diabetes Mellitus Through a Metabolism-Independent Pathway. J. Am. Heart Assoc..

[79] During M.J., Cao L., Zuzga D.S., Francis J.S., Fitzsimons H.L., Jiao X., Bland R.J., Klugmann M., Banks W.A., Drucker D.J. (2003). Glucagon-like peptide-1 receptor is involved in learning and neuroprotection. Nat. Med..

[80] Tuulari J.J., Karlsson H.K., Hirvonen J., Salminen P., Nuutila P., Nummenmaa L. (2015). Neural circuits for cognitive appetite control in healthy and obese individuals: An fMRI study. PLoS ONE.

[81] Volkow N.D., Wang G.-J., Tomasi D., Baler R.D. (2013). The addictive dimensionality of obesity. Biol. Psychiatry.

[82] Rösch S.A., Schmidt R., Lührs M., Ehlis A.-C., Hesse S., Hilbert A. (2020). Evidence of fNIRS-Based Prefrontal Cortex Hypoactivity in Obesity and Binge-Eating Disorder. Brain Sci..

[83] Deng Z., Huang Q., Huang J., Zhang W., Qi C., Xu X. (2017). Association between central obesity and executive function as assessed by stroop task performance: A functional near-infrared spectroscopy study. J. Innov. Opt. Health Sci..

[84] Veit R., Schag K., Schopf E., Borutta M., Kreutzer J., Ehlis A.-C., Zipfel S., Giel K.E., Preissl H., Kullmann S. (2021). Diminished prefrontal cortex activation in patients with binge eating disorder associates with trait impulsivity and improves after impulsivity-focused treatment based on a randomized controlled IMPULS trial. NeuroImage Clin..

[85] Xu X., Deng Z.-Y., Huang Q., Zhang W.-X., Qi C.-Z., Huang J.-A. (2017). Prefrontal cortex-mediated executive function as assessed by Stroop task performance associates with weight loss among overweight and obese adolescents and young adults. Behav. Brain Res..

[86] Murdaugh D.L., Cox J.E., Cook E.W., Weller R.E. (2012). fMRI reactivity to high-calorie food pictures predicts short- and long-term outcome in a weight-loss program. Neuroimage.

[87] Huang J., Xiong M., Xiao X., Xu X., Hong X. (2019). fNIRS correlates of the development of inhibitory control in young obese subjects. J. Integr. Neurosci..

[88] Schroeter M.L., Zysset S., Wahl M., von Cramon D.Y. (2004). Prefrontal activation due to Stroop interference increases during development--an event-related fNIRS study. Neuroimage.

[89] Ljubisavljevic M., Maxood K., Bjekic J., Oommen J., Nagelkerke N. (2016). Long-Term Effects of Repeated Prefrontal Cortex Transcranial Direct Current Stimulation (tDCS) on Food Craving in Normal and Overweight Young Adults. Brain Stimul..

[90] Luzi L., Gandini S., Massarini S., Bellerba F., Terruzzi I., Senesi P., Macri C., Ferrulli A. (2021). Reduction of impulsivity in patients receiving deep transcranial magnetic stimulation treatment for obesity. Endocrine.

[91] Cox D., Gonder-Frederick L., McCall A., Kovatchev B., Clarke W. (2002). The effects of glucose fluctuation on cognitive function and QOL: The functional costs of hypoglycaemia and hyperglycaemia among adults with type 1 or type 2 diabetes. Int. J. Clin. Pract. Suppl..

[92] Service F.J., Molnar G.D., Rosevear J.W., Ackerman E., Gatewood L.C., Taylor W.F. (1970). Mean amplitude of glycemic excursions, a measure of diabetic instability. Diabetes.

[93] Rizzo M.R., Marfella R., Barbieri M., Boccardi V., Vestini F., Lettieri B., Canonico S., Paolisso G. (2010). Relationships between daily acute glucose fluctuations and cognitive performance among aged type 2 diabetic patients. Diabetes Care.

[94] Giordani I., Di Flaviani A., Picconi F., Malandrucco I., Ylli D., Palazzo P., Altavilla R., Vernieri F., Passarelli F., Donno S. (2014). Acute hyperglycemia reduces cerebrovascular reactivity: The role of glycemic variability. J. Clin. Endocrinol. Metab..

[95] Huang J., Zhang X., Li J., Tang L., Jiao X., Lv X. (2014). Impact of glucose fluctuation on acute cerebral infarction in type 2 diabetes. Can. J. Neurol. Sci..

[96] Kurtz P., Claassen J., Helbok R., Schmidt J., Fernandez L., Presciutti M., Stuart R.M., Connolly E.S., Lee K., Badjatia N. (2014). Systemic glucose variability predicts cerebral metabolic distress and mortality after subarachnoid hemorrhage: A retrospective observational study. Crit. Care.

[97] Santana D., Mosteiro A., Pedrosa L., Llull L., Torné R., Amaro S. (2022). Clinical relevance of glucose metrics during the early brain injury period after aneurysmal subarachnoid hemorrhage: An opportunity for continuous glucose monitoring. Front. Neurol..

[98] Sugimoto T., Tokuda H., Miura H., Kawashima S., Ando T., Kuroda Y., Matsumoto N., Fujita K., Uchida K., Kishino Y. (2023). Cross-sectional association of metrics derived from continuous glucose monitoring with cognitive performance in older adults with type 2 diabetes. Diabetes Obes. Metab..

[99] Matsubara M., Makino H., Washida K., Matsuo M., Koezuka R., Ohata Y., Tamanaha T., Honda-Kohmo K., Noguchi M., Tomita T. (2020). A Prospective Longitudinal Study on the Relationship Between Glucose Fluctuation and Cognitive Function in Type 2 Diabetes: PROPOSAL Study Protocol. Diabetes Ther..

[100] Li W., Maloney R.E., Aw T.Y. (2015). High glucose, glucose fluctuation and carbonyl stress enhance brain microvascular endothelial barrier dysfunction: Implications for diabetic cerebral microvasculature. Redox Biol..

[101] Cardoso S., Seiça R.M., Moreira P.I. (2018). Uncoupling Protein 2 Inhibition Exacerbates Glucose Fluctuation-Mediated Neuronal Effects. Neurotox. Res..

[102] Lv Y., Zhu L.-L., Shu G.-H. (2018). Relationship between Blood Glucose Fluctuation and Brain Damage in the Hypoglycemia Neonates. Am. J. Perinatol..

[103] Meng Q.-Z., Wang Y., Li B., Xi Z., Wang M., Xiu J.-Q., Yang X.-P. (2023). Relationship between glycemic variability and cognitive function in lacune patients with type 2 diabetes. World J. Clin. Cases.

[104] Diéguez E., Nieto-Ruiz A., Martín-Pérez C., Sepúlveda-Valbuena N., Herrmann F., Jiménez J., De-Castellar R., Catena A., Garcia-Santos J.A., Bermudez M.G. (2022). Association study between hypothalamic functional connectivity, early nutrition, and glucose levels in healthy children aged 6 years: The COGNIS study follow-up. Front. Nutr..

[105] Araki S., Yamamoto Y., Saito R., Kawakita A., Eguchi M., Goto M., Kubo K., Kawagoe R., Kawada Y., Kusuhara K. (2017). Plasma but not serum brain-derived neurotrophic factor concentration is decreased by oral glucose tolerance test-induced hyperglycemia in children. J. Pediatr. Endocrinol. Metab..

[106] Lee I.-T., Chen C.-H., Wang J.-S., Fu C.-P., Lee W.-J., Liang K.-W., Lin S.-Y., Sheu W.H.-H. (2018). The association between brain-derived neurotrophic factor and central pulse pressure after an oral glucose tolerance test. Clin. Chim. Acta.

[107] Lee I.-T., Li Y.-H., Sheu W.H.-H. (2020). Brain-Derived Neurotrophic Factor during Oral Glucose Tolerance Test Predicts Cardiovascular Outcomes. Int. J. Mol. Sci..

[108] Maric G., Lalic K., Pekmezovic T., Tamas O., Rajkovic N., Rasulic I., Mesaros S., Drulovic J. (2020). Could the performance of oral glucose tolerance test contribute to the brain health-focused care in multiple sclerosis?. Mult. Scler. Relat. Disord..

